# The Role of Crk Adaptor Proteins in T-Cell Adhesion and Migration

**DOI:** 10.3389/fimmu.2015.00509

**Published:** 2015-10-05

**Authors:** Alex Braiman, Noah Isakov

**Affiliations:** ^1^The Shraga Segal Department of Microbiology, Immunology and Genetics, Faculty of Health Sciences, The Cancer Research Center, Ben Gurion University of the Negev, Beer Sheva, Israel; ^2^School of Pharmacy, University of Otago, Dunedin, New Zealand

**Keywords:** Crk adaptor proteins, signal transduction, T lymphocytes, cell adhesion, cell migration, immunophilins, cyclophilin A, cyclosporin A

## Abstract

Crk adaptor proteins are key players in signal transduction from a variety of cell surface receptors. They are involved in early steps of lymphocyte activation through their SH2-mediated transient interaction with signal transducing effector molecules, such as Cbl, ZAP-70, CasL, and STAT5. In addition, they constitutively associate, via their SH3 domain, with effector molecules, such as C3G, that mediate cell adhesion and regulate lymphocyte extravasation and recruitment to sites of inflammation. Recent studies demonstrated that the conformation and function of CrkII is subjected to a regulation by immunophilins, which also affect CrkII-dependent T-cell adhesion to fibronectin and migration toward chemokines. This article addresses mechanisms that regulate CrkII conformation and function, in general, and emphasizes the role of Crk proteins in receptor-coupled signaling pathways that control T-lymphocyte adhesion and migration to inflammatory sites.

## Introduction

Adaptor proteins are essential components of signal transduction pathways in all cell types due to their ability to link engaged receptors to specific downstream signaling cascades. The presence of multiple protein–protein interaction domains in an adaptor protein enables the simultaneous interaction with two or more effector molecules and the spatially and temporally orchestrated assembly of multimolecular complexes that are essential for signal transduction.

The mammalian CT10 (chicken tumor virus number 10) regulator of kinase (Crk) adaptor proteins, CrkI and CrkII, are alternatively spliced products of a single gene (1). Both proteins include an N-terminal Src homology 2 (SH2) domain followed by a C-terminal SH3 domain (2–4). CrkII includes an additional SH3 domain at the C-terminus (SH3C), which is separated from the SH3N by a 47-residue-containing spacer region. A third member of the family, termed Crk-like (CrkL), is encoded by a closely related gene and possesses an overall structure similar to that of CrkII (2–4).

The Crk proteins are ubiquitously expressed and act as major convergence points of tyrosine kinase signaling pathways. They utilize their SH2 and SH3 domains for interaction with multiple effector molecules and integrate molecular information obtained from diverse sources, such as growth factors, extracellular matrix (ECM), pathogens, and apoptotic cells (2–6). In general, Crk proteins play an essential role in the regulation of immune cell functions leading to cell adhesion and migration, proliferation, differentiation, and apoptosis (2–6).

## The Structure and Binding Partners of Crk

**Figure 1:**
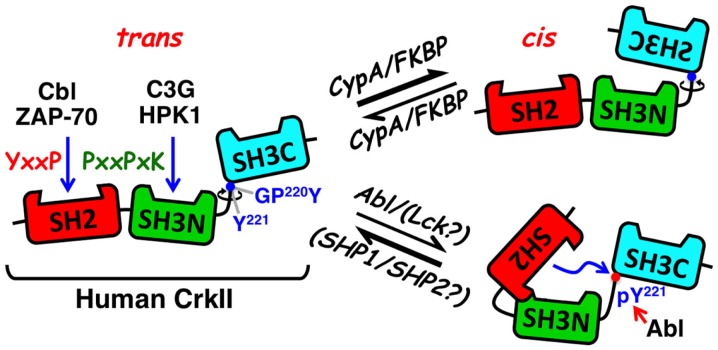
**Schematic structure and mode of regulation of CrkII**. The CrkII protein contains two protein–protein interaction domains and a single regulatory domain. The N-terminal CrkII-SH2 domain (red) transiently interacts with phosphorylated tyrosine-containing peptide sequences possessing a pY-x-x-P consensus motif, which is found in signaling proteins, such as tyrosine-phosphorylated Cbl and ZAP-70. The CrkII-SH3N domain (green) interacts with polyproline-rich sequences in proteins such as C3G and HPK1, with a preference toward the P-x-L-P-x-K motif. The CrkII-SH3C (light blue) is an atypical SH3 domain due to the presence of hydrophobic, instead of aromatic, residues in its polyproline binding pocket, which reduces its affinity to polyproline-containing peptides. It functions predominantly as a regulatory region, which under certain conditions can block ligand interaction with CrkII. A 47-residue-containing spacer region, which links the two SH3 domains, contains a tyrosine residue (Tyr 221) that is a target for phosphorylation by Abl, in ligand-stimulated cells. Phospho-Tyr221 can intramolecularly interact with, and block the SH2 domain, and induce a conformational change that alters CrkII accessibility to other potential ligands. A second motif in the linker region is a substrate for the peptidyl-prolyl *cis–trans* isomerases (PPIases), such as cyclophilin A and FK506, which can catalyze the *cis*–*trans* isomerization of the Gly-Pro^220^ peptide bond and interconverts CrkII between two distinct conformations. The CrkII *cis* isomer adopts a closed, autoinhibited conformation, while the CrkII *trans* isomer adopts an open, uninhibited conformation, which is available for interaction with SH2 and SH3 binding partners. Blue arrows indicate sites of interaction and a red arrow indicates a site of phosphorylation. Abl, Abelson murine leukemia viral oncogene; C3G, Cbl, Casitas B-lineage lymphoma; CRK, SH3 domain-binding guanine nucleotide-releasing factor; Crk, CT10 (chicken tumor virus number 10) regulator of kinase; CypA, cyclophilin A; FKBP, FK506 binding protein; GPY, Gly-Pro-Tyr motif; HPK1, hematopoietic progenitor kinase 1; Lck, lymphocyte-specific protein tyrosine kinase; pY, phosphotyrosine; SH2, Src homology 2; SH3C, C-terminal Src homology domain 3; SH3N, N-terminal Src homology domain 3; PPIase, peptidyl-prolyl *cis–trans* isomerase; SHP1, SH2 domain-containing protein tyrosine phosphatase 1; SHP2, SH2 domain-containing protein tyrosine phosphatase 2; Y, tyrosine residue; ZAP-70, zeta-chain-associated protein kinase 70.

**Figure 2:**
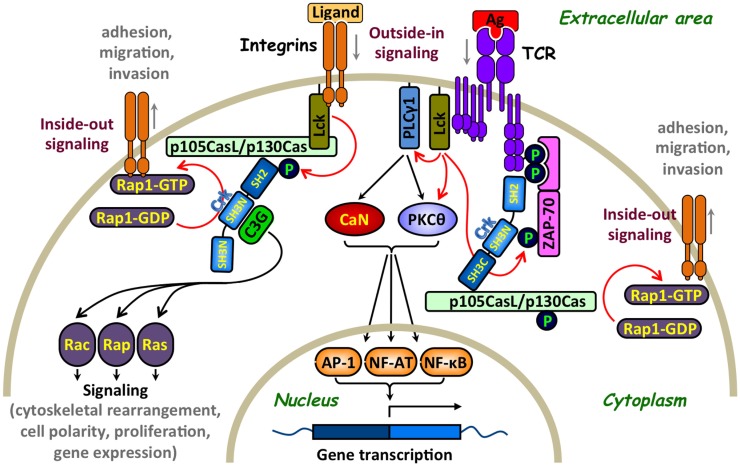
**Involvement of Crk adaptor proteins in TCR- and integrin-coupled signaling pathways that regulate T-lymphocyte adhesion**. A simplified scheme of surface receptors and signaling pathways that regulate T-cell activation and adhesion. T-cell receptor (TCR) ligation results in Lck-mediated tyrosine phosphorylation of immunoreceptor tyrosine-based activation motifs (ITAMs) on the intracellular portion of the CD3 subunits, leading to the recruitment of ζ-chain-associated protein kinase of 70 kDa (ZAP-70) and its phosphorylation on several distinct tyrosine residues, including Tyr315 (13). The subsequent Lck- and ZAP-70-mediated tyrosine phosphorylation of multiple substrates activates several signaling cascades, in which calcineurin (CaN) and protein kinase C theta (PKCθ) play a major role, leading to cell activation, proliferation, and differentiation. In parallel, Crk adaptor proteins transiently interact via their SH2 domain with ZAP-70 Tyr315 and, apparently, promote the recruitment of additional Crk-binding proteins to the receptor site that contribute to the “outside-in” signaling pathway (11). Crk interaction with the guanine nucleotide-exchange factor (GEF), C3G, imposes the conversion of the small GTPase, RAP1, to an active GTP-bound protein, which in turn leads to activation of the LFA-1 integrin and increases T-cell adhesion. Activation of this “inside-out” signaling pathway, which promotes integrin-mediated T-cell adhesion, is sensitive to a regulation by immunophilins (14). GEF-mediated activation of Rap1, which is essential for upregulation of the integrin receptors, can also be mediated by a WASP family verprolin-homologous protein-2 (WAVE2)-regulated CrkL-C3G complexes (15) as well as by other Crk-independent pathways (16). Integrins can also initiate “outside-in” and “inside-out” signaling pathways since their interaction with ICAM-1 or extracellular matrix results in signal delivery that promotes conformational changes in adjacent integrins leading to increase in their clustering and binding affinity. For simplicity, additional effector proteins that interact with Crk, such as WAVE and immunophilins, which are also involved in the regulation of T-cell adhesion, are not included in the figure. Red arrows indicate direct effects and black arrows indicate indirect effects. Ag, antigen; AP-1, activation protein-1; C3G, CRK SH3 domain-binding guanine nucleotide-releasing factor; CaN, calcineurin; Cas, Crk-associated substrate; CasL, Cas lymphocyte type; Crk, CT10 (chicken tumor virus number 10) regulator of kinase; GDP, guanosine diphosphate; GTP, guanosine-5′-triphosphate; Lck, lymphocyte-specific protein tyrosine kinase; NF-AT, nuclear factor of activated T cells; NF-κB, nuclear factor kappa-light-chain-enhancer of activated B cells; P, a site of tyrosine phosphorylation; PKCθ, protein kinase C theta; PLCγ1, phospholipase C gamma 1; Rac, Ras-related C3 botulinum toxin substrate; Rap, Ras-related protein; Ras, rat sarcoma; ZAP-70, zeta-chain-associated protein kinase 70.

Crk proteins possess two types of basic modules that function as protein–protein interaction domains. The Crk amino-terminus SH2 domain binds tyrosine-phosphorylated proteins containing the preferred consensus motif, pY-x-x-P, whereby specificity is provided by the two to five residues C-terminal of the phosphotyrosyl group (7) (Figure 1). In accordance, Crk proteins interact via their SH2 domain with a large number of T-lymphocyte effector proteins, including the Crk-associated substrate lymphocyte type [p105CasL, also known as human enhancer of filamentation protein 1 (HEF1) or NEDD9] (8), Casitas B-lineage lymphoma (c-Cbl) (9, 10), and ζ-chain-associated protein kinase 70kDa (ZAP-70) (11, 12) (Figure 2).

A second module found in all Crk proteins is an SH3 domain, which binds polyproline-rich sequences, with a preference toward the P-x-L-P-x-K motif (17) (Figure 1). In T cells, this domain interacts with proteins such as the Crk SH3N domain-binding guanine nucleotide-releasing factor (C3G) (9, 18) and the hematopoietic progenitor kinase 1 (HPK1) (19, 20) (Figure 2). It has been suggested that the CrkI/II- and CrkL-SH3N domains exhibit redundant substrate specificity and constitutively interact with their ligands, independent of the cell activation state (21, 22). Both SH2 and SH3 domains of CrkII interact with multiple binding partners in different cell types (2–6), although information about such interactions in T cells is relatively limited.

The SH3C of both CrkII and CrkL possesses a consensus SH3 sequence homology, but its binding surface contains some hydrophobic residues that substantially reduce its affinity to polyproline-rich sequences (23). Two independent studies have indicated that the Crk-SH3C domain contains a nuclear export signal that interacts with the Crm1/Exportin (24, 25). However, the physiological relevance of this low-affinity interaction has yet to be determined.

More recent studies suggested that the main function of the SH3C domain is directed at stabilizing CrkII and/or CrkL in a conformation that negatively regulates their biological function (25–29).

## Conformation, Function, and Expression Regulation of Crk Adaptor Proteins

Apart from the SH2 and SH3 domains, Crk proteins contain additional regulatory motifs that may have an impact on the protein conformation and biological activity (Figure 1). One such motif includes a tyrosine residue within the linker region tethering the two SH3 domains (Tyr221 and Tyr207 in the human CrkII and CrkL, respectively) (30, 31). This tyrosine undergoes phosphorylation by a protein tyrosine kinase (PTK), such as Abl, following the interaction of a number of different ligands with their cognate cell surface receptors (31, 32). In addition, Tyr207 in CrkL is intensely phosphorylated by the oncogenic tyrosine kinase Bcr-Abl in Philadelphia chromosome-positive leukemia (30, 32), where CrkL is required for the Bcr-Abl-induced cell transformation (33).

The conserved tyrosine residues in CrkII and CrkL reside within a Y-x-x-P sequence, and upon phosphorylation, they internally interact with the SH2 domain, thus operating as dominant-negative binding motifs (32). NMR spectrometry analysis confirmed that phospho-Tyr221 in CrkII does indeed interact with the adjacent CrkII-SH2 domain and alters the protein’s structure (34). The ensuing conformational change is expected to block the SH3N, in addition to the SH2 domain, releasing CrkII from its binding partners and repressing CrkII-mediated signaling (32, 34).

A second regulatory motif within an extended loop in the SH2 domain possesses a proline-rich region, which is dispensable for phosphopeptide binding and is able to interact with various SH3 domains, including that of the Abl PTK (32, 35, 36). Although the *in vitro* interaction of the full-length non-phosphorylated CrkII with Abl SH3 domain was rather weak, phosphorylation of CrkII-Tyr221, or addition of a phosphopeptide corresponding to the CrkII-Tyr221 phosphorylation site, stimulated CrkII association with the Abl SH3 domain. The results suggested that the proline-rich insert within the Crk SH2 domain constitutes an SH3 domain-binding motif which is regulated by phosphopeptide ligand binding to the Crk SH2 domain. CrkI possesses an identical SH3N domain allowing it to associate with Abl, but due to the lack of the linker region that includes Tyr221 (in addition to the entire SH3C domain), it cannot undergo phosphorylation *in vivo*, and apparently exhibits a very low affinity to the Abl SH3 domain.

Recent studies revealed an additional regulatory motif within the linker region of the chicken, but not human, CrkII, which serves as a target for peptidyl-prolyl *cis–trans* isomerases (PPIases) (28, 29, 37, 38). Interconversion between the *cis* and *trans* isomers of the chicken CrkII occurred at the Gly237-Pro238 prolyl bond and was catalyzed by the PPIase, cyclophilin A (CypA). Acquisition of a *cis* conformation resulted in an intramolecular interaction between the two SH3 domains of CrkII, forming a closed conformation of an autoinhibited molecule. In contrast, the CrkII *trans* conformer acquired a relatively open and extended conformation that was readily available for interaction with SH2- and SH3N-binding partners. PPIases catalyze both *cis-*to-*trans* and *trans*-to-*cis* interconversion of CrkII, thereby determining the temporal and spatial activation of CrkII and CrkII-regulated signaling events.

More recent studies demonstrated that human T-cell-derived CrkII, but not CrkI, can physically interact with representatives of two families of PPIases, CypA and FK506 binding protein (FKBP), collectively termed immunophilins (14). *In vitro* coincubation of CypA with human T-cell-derived CrkII increased CrkII binding to C3G while immunophilin inhibitors [such as cyclosporine A (CsA) and FK506] downregulated CrkII binding to C3G, suggesting that CrkII-C3G interaction may be subjected to a regulation by immunophilins (14). This assumption was supported by fluorescence resonance energy transfer (FRET) studies of Jurkat T cells transfected with PICCHUx, a chimeric plasmid encoding the human CrkII_1-236_ sandwiched between cyan fluorescent protein (CFP) and yellow fluorescent protein (YFP). The basal FRET level of these cells was further augmented by CsA and FK506 treatment, reflecting a drug-induced inhibition of immunophilins and a *trans*-to-*cis* conversion of CrkII.

The differential sensitivity of the truncated chicken vs. human CrkII to PPIases was attributed to the nature of the amino acid that immediately follows the Pro238 (chicken)/237 (human) residue, Phe and Ile in the chicken and the human CrkII, respectively (28, 29, 38). Since PICCHUx encodes a truncated human CrkII, which lacks Pro237, we predict that the Gly219-Pro220 motif plus its adjacent Tyr221 residue (an aromatic amino acid that is structurally related to Phe, except for a singly extra hydroxyl group) may be the site of immunophilin-mediated isomerization of the human CrkII.

CrkII shares with CrkL a high degree of sequence homology and ligand-binding preference, but the two proteins have distinct physiological roles. Structural studies demonstrated that the two proteins exhibit a distinct intramolecular assembly of their domains (39, 40), which might explain the differences in their biological activities. Furthermore, CrkL, unlike CrkII, lacks an immunophilin substrate motif.

Another line of studies demonstrated that the extent of expression of Crk is subjected to a regulation by microRNA (miRNA) (41). These studies were based on bioinformatic analyses predicting that Crk can be directly targeted by miR-126. Indeed, analysis performed in HSC-T6 cells (an immortalized rat liver stellate cell line) revealed that the Crk 3′UTR is a direct target for miR-126. Furthermore, miR-126 overexpression led to decreased Crk protein expression, suggesting that Crk function and Crk-dependent signaling pathways may also be regulated at the Crk protein expression level.

## Crk-Mediated Regulation of T-Cell Adhesion and Motility

The ability of T lymphocytes to physically interact with antigen-presenting cells (APCs) and adhere to vascular endothelial cells is absolutely essential for the induction of all types of T-cell-mediated immune responses. Accordingly, deficiency in adhesion molecules and/or their signaling pathways, such as in leukocyte adhesion deficiency (LAD), results in impaired or complete absence of immune responses (42). Interaction of T cells with APCs is a prerequisite for antigen recognition by the T-cell antigen receptor (TCR) and the subsequent activation and differentiation of the T cells into functional effector cells (43). In addition, migration of T cells to sites of inflammation is dependent on their ability to adhere to endothelial cells as the first step of extravasation and subsequent migration (44). Adhesion of T cells is mediated by members of the integrin family of receptors, such as lymphocyte function-associated antigen 1 (LFA-1; CD11a/CD18) and very late antigen-4 (VLA-4, α4β1-integrin), and additional receptors, such as l-selectins, CD44, and CD31 (44).

A large number of T-cell effector molecules play a role in the transduction of signals regulating the activity of adhesion receptors (15, 45–47). C3G is the first protein identified as being associated with Crk in a constitutive manner and is involved in cell adhesion (48, 49). It catalyzes guanosine-5′-triphosphate (GTP) exchange by the small G-protein that removes GDP from small GTPases, such as Rap-1, Rap-2, and R-Ras, allowing their spontaneous reloading with GTP (50). These findings were initially observed in fibroblasts or epithelial cells and later on confirmed in other cell types, including lymphocytes. The targets of Rap-1 have been implicated in cell proliferation, cytoskeletal reorganization, and cell-to-cell contact (50–52). One of the most important biological effects of the Crk-C3G-Rap1 signaling pathway is the regulation of integrins, which mediate adherence of hematopoietic cells to ECM and stromal cells. The recruitment of C3G to Crk and subsequent loading of Rap1 with GTP increases the affinity of β1-integrins to the ECM (51, 53). This CrkL–C3G complex is required for a TCR-coupled signaling pathway, which involves the WASP family verprolin-homologous protein-2 (WAVE2) protein and delivers signals that activate Rap1, regulate both clustering and affinity maturation of integrins, and promote integrin-dependent cell adhesion (15). In addition, overexpressed CrkL was found to increase cell adhesion to fibronectin, a process that requires a functional CrkL-SH3N domain (54, 55).

The CasL docking protein (56) is linked to the β1-integrin signaling which provides a costimulus for TCR/CD3-driven T-cell proliferation (57). T cells expressing low levels of CasL exhibited impaired β1-integrin-mediated costimulation, a defect that was restored by cell transfection of CasL (58). In TCR-activated T cells, CasL undergoes tyrosine phosphorylation at multiple Y-D-x-P motifs, which serve high-affinity docking sites for Crk-SH2 domains (8, 47, 58). The CasL–Crk complex, in turn, recruits the guanine nucleotide exchange factor (GEF), C3G, which effectively catalyzes the guanine nucleotide exchange reaction for Rap1 (21).

The important role of Crk in coordinating C3G-CasL-Rap1 signals leading to integrin-dependent T-cell adhesion and migration was further substantiated by analysis of T cells from *Crk* and *CrkL* gene conditional knockout mice (21). These T cells were impaired in their ability to activate Rap1 and responded to chemokines by increased adhesion and migration to sites of inflammation.

While studies on lymphocytes emphasized the role of Crk in the regulation of the C3G-dependent activation of Rap1, other studies demonstrated that Crk regulates additional Rho family GTPases (59–61) and involved in the regulation of cell migration by coordinating stimulus-dependent reorganization of the actin cytoskeleton (61, 62).

Recent studies demonstrated that the Abl kinases, as well as the CasL and Hef1-associated signal transducer in hematopoietic cells (Chat-H), are integral components of the signaling pathway that promotes Rap1 activation and chemokine-induced T-cell migration (63, 64). Abl kinases, Abl and Arg, are known to be activated in response to TCR engagement and contribute to maximal TCR signaling (65). In addition, they are activated downstream of the SDF-1α chemokine receptor and control the tyrosine phosphorylation of CasL, an upstream regulator of Rap1 (63). Thus, by coupling the regulation of CasL and Rap1 effector proteins to chemokine receptors, the Abl/Arg kinases link chemokine signals to T-cell polarization and migration. In agreement with the above findings, loss of Abl and Arg in mouse T cells inhibited their homeostatic migration and ability to recruit to sites of inflammation (63).

The Chat-H is a member of the novel SH2-containing protein (NSP) family (66), also known as the hematopoietic organ-specific NSP3 isoform. It operates upstream of Rap1 and is required for both basal and chemokine-induced phosphorylation of CasL. Chat-H-depleted T cells exhibited diminished chemokine-induced activation of Rap1 that coincided with impaired integrin-mediated adhesion and migration. It is therefore assumed that the effector function of Chat-H involves the coupling of the chemokine receptor signaling pathways to integrin-mediated T-cell adhesion (64).

The ZAP-70 PTK is a Crk-binding protein that critically involved in TCR signaling and also regulates integrin receptor function (67–71). TCR stimulation of ZAP-70-negative T cells failed to induce β1-integrin-dependent T-cell adhesion to fibronectin, a defect that was corrected by the reintroduction of ZAP-70 to the cells (67). In addition, TCR activation-dependent binding of the lymphocyte function-associated antigen 1 (LFA-1) integrin to its ligand, intercellular adhesion molecule 1 (ICAM-1), initiates the delivery of “inside-out” signals to the LFA-1 in a ZAP-70-dependent manner (69). In these studies, overexpression of a dominant-negative ZAP-70 in a T-cell hybridoma blocked the LFA-1-dependent *in vitro* invasion of the hybridoma cells, inhibited their ability to disseminate *in vivo* and form remote metastases, and suppressed their ability to migrate toward the chemokine, stromal cell-derived factor 1 (SDF-1) (69). ZAP-70 associates both with the TCR and LFA-1 receptors (68, 70, 72) and is essential for the delivery of “outside-in” and “inside-out” signals leading, among other things, to T-cell adhesion. Since Crk proteins interact with tyrosine phosphorylated and activated ZAP-70 in a T-cell activation-dependent manner (11, 12, 73), the physical interaction of ZAP-70 with Crk might be required for the coupling of ZAP-70 to effector molecules operating downstream of activated TCR and/or integrin receptors. In such a case, TCR activation-dependent Crk binding to ZAP-70 might contribute both to “outside-in” and “inside-out” signals that upregulate integrin-mediated T-cell adhesion. This model is partially supported by findings from two independent studies, showing that ZAP-70 with a Phe-to-Tyr substitution at position 315 abolishes both CrkII binding to ZAP-70 (11) and TCR activation-dependent increase in β1-integrin-mediated cell adhesion (71). Further studies with Crk-SH2 mutants deficient in their ability to interact with ZAP-70 are needed in order to resolve this issue.

As described earlier, one of the modes of regulation of CrkII conformation and function involves its *cis–trans* isomerization controlled by immunophilins (28, 29, 37). Our studies revealed that a combination of immunophilin inhibitory drugs, which includes CsA and FK506, interferes with C3G binding to CrkII (14). This, in turn, impaired the integrin-associated signaling and inhibited T-cell adhesion to fibronectin as well as migration toward the chemokine, SDF-1α (14). We found that overexpression of either CrkI or CrkII increased T-cell adhesion and invasion. However, inhibitors of immunophilin suppressed the ability of CrkII-, but not CrkI-overexpressing Jurkat T cells to adhere to fibronectin-coated surfaces and migrate toward SDF-1α (14).

An additional effector molecule that regulates T-cell adhesion by alteration of Crk-dependent signaling pathways is the E3-ligase, Cbl (74). The Cbl protein catalyzes protein ubiquitination and is involved in the regulation of TCR-coupled signals that induce T-cell adhesion. Upon activation of T cells, Cbl undergoes tyrosine phosphorylation and associates with the Crk-SH2 domain and p85, the regulatory subunit of the phosphoinositide 3-kinase (PI3-K), forming a trimolecular protein complex (74). Cbl-b binding to CrkL induces the ubiquitination of CrkL, while knockdown of Cbl-b results in an increase in several parameters relevant to T-cell adhesion, including the association of CrkL and C3G, activity of the small GTPase, Rap1, and clustering of LFA-1, in response to TCR triggering (75, 76). The results suggest that Cbl-b is a negative regulator of the CrkL-C3G-mediated Rap1 and LFA-1 activation of T cells and of Crk-regulated signaling events that promote T-cell adhesion (75, 76). Protein complexes consisting of Crk and Cbl are also involved in signaling from the l-selectin (CD62) adhesion receptors (77) as well as the CXCR4 chemokine receptor (78). Taken together, the results suggest that Cbl-mediated regulation of Crk-dependent signals is an important element in the regulation of T-lymphocyte adhesion, migration, and homing (79).

## Concluding Remarks and Future Directions

The Crk adaptor proteins play an essential role in the regulation of T-cell adhesion and migration to sites of inflammation. Together with C3G and CasL, they coordinate signaling pathways leading to activation of Rap1 that upregulate the clustering and affinity of integrins to their ligands. This general mechanism might be relevant to the regulation of some, but not all, integrins since different T-cell integrins might be coupled to distinct effector proteins. The mechanism of regulation of the stoichiometric ratio between CrkI, CrkII, and CrkL in specific cell types is unknown, and the contribution of individual Crk isoforms to the adhesion response has not been thoroughly studied. Further investigations are also required in order to determine the potential regulation of selected Crk proteins by isomerases and whether inhibitors of immunophilins can downregulate T-cell adhesion and migration irrespective of their inhibition of the calcineurin/NFAT pathway.

## Conflict of Interest Statement

The authors declare that the research was conducted in the absence of any commercial or financial relationships that could be construed as a potential conflict of interest.
